# Keratin 19 binds and regulates cytoplasmic HNRNPK mRNA targets in triple-negative breast cancer

**DOI:** 10.1186/s12860-023-00488-z

**Published:** 2023-08-17

**Authors:** Arwa Fallatah, Dimitrios G. Anastasakis, Amirhossein Manzourolajdad, Pooja Sharma, Xiantao Wang, Alexis Jacob, Sarah Alsharif, Ahmed Elgerbi, Pierre A. Coulombe, Markus Hafner, Byung Min Chung

**Affiliations:** 1 Department of Biology, The Catholic University of America, Washington, DC United States of America; 2 RNA Molecular Biology Laboratory, National Institute of Arthritis and Musculoskeletal and Skin Diseases, Bethesda, MD United States of America; 3 Department of Cell and Developmental Biology, University of Michigan Medical School, Ann Arbor, MI United States of America; 4 Department of Dermatology, University of Michigan Medical School, Ann Arbor, MI United States of America; 5 Present Address: Department of Computer Science, Colgate University, Hamilton, NY United States of America

**Keywords:** Keratin 19, HNRNPK, PAR-CLIP, Gene expression, Breast Cancer, p53, Proliferation, RNA binding protein, RNA-protein interaction

## Abstract

**Background:**

Heterogeneous nuclear ribonucleoprotein K (HNRNPK) regulates pre-mRNA processing and long non-coding RNA localization in the nucleus. It was previously shown that shuttling of HNRNPK to the cytoplasm promotes cell proliferation and cancer metastasis. However, the mechanism of HNRNPK cytoplasmic localization, its cytoplasmic RNA ligands, and impact on post-transcriptional gene regulation remain uncharacterized.

**Results:**

Here we show that the intermediate filament protein Keratin 19 (K19) directly interacts with HNRNPK and sequesters it in the cytoplasm. Correspondingly, in K19 knockout breast cancer cells, HNRNPK does not localize in the cytoplasm, resulting in reduced cell proliferation. We comprehensively mapped HNRNPK binding sites on mRNAs and showed that, in the cytoplasm, K19-mediated HNRNPK-retention increases the abundance of target mRNAs bound to the 3’ untranslated region (3’UTR) at the expected cytidine-rich (C-rich) sequence elements. Furthermore, these mRNAs protected by HNRNPK in the cytoplasm are typically involved in cancer progression and include the p53 signaling pathway that is dysregulated upon HNRNPK knockdown (HNRNPK KD) or K19 knockout (*KRT19* KO).

**Conclusions:**

This study identifies how a cytoskeletal protein can directly regulate gene expression by controlling the subcellular localization of RNA-binding proteins to support pathways involved in cancer progression.

**Supplementary Information:**

The online version contains supplementary material available at 10.1186/s12860-023-00488-z.

## Background

Heterogeneous nuclear ribonucleoproteins (HNRNPs) are a group of abundant nuclear RNA binding proteins (RBPs) consisting of more than 20 members from various RBP families that predominantly localize to the cell nucleus [1]. One of the HNRNPs that promotes tumorigenesis is HNRNPK [2]. It comprises three K-homology (KH) RNA binding domains with a clear preference for C-rich sequences [3], in addition to a less characterized K interactive region (KI) that is thought to allow interaction with multiple other proteins [1]. HNRNPK plays crucial roles in several biological processes, including development, axonal outgrowth, cell proliferation, and migration [4–7]. In cancer, overexpression of HNRNPK promotes tumor progression and correlates with poor patient survival [8], likely by directly affecting the expression and activities of oncogenes and tumor suppressors such as EIF4E [9], c-MYC [10], c-SRC [11] and MDM2 [12]. As the list of oncogenes and tumor suppressors regulated by HNRNPK is still growing, a comprehensive perspective of HNRNPK-target genes and their regulatory mechanisms remain elusive. Considering that KH domains typically only recognize a few nucleotides in RNA [13], systems-wide methods are required for identifying HNRNPK-controlled posttranscriptional regulatory networks. While a few studies used crosslinking and immunoprecipitation (CLIP)-type approaches to catalog HNRNPK target mRNAs [14, 15], its genome-wide regulatory impact has not yet been determined.

While HNRNPK primarily resides in the nucleus [1], it impacts all steps of gene expression and a cytoplasmic pool of HNRNPK has been linked to metastasis and poor prognosis in cancer [3, 16, 17]. HNRNPK contains a nuclear shuttling domain [18], and posttranslational modifications of this domain, such as phosphorylation by ERK kinase [19] and methylation by PRMT1 [19] contribute to the regulation of the subcellular localization of HNRNPK. A previous study in skin cancer cells showed that a cytoskeletal protein keratin 17 (K17) is required for the cytoplasmic localization of HNRNPK [4], demonstrating that keratin is a regulator of HNRNPK localization.

The keratin family is composed of 54 genes, and the expression of each keratin is tightly regulated in a tissue-, context-, and differentiation-specific manner. Among keratins, keratin 19 (K19) is the smallest with a very short tail domain. It is expressed in various simple and complex epithelial tissues and becomes upregulated in several cancers where it is used as a diagnostic and prognostic marker [20] Altered expression of K19 affects the growth of cancer cells in vitro and tumors in mice [20, 21], demonstrating its active role in cancer.

Inside the cell, keratins can interact with multiple proteins and regulate their localization. K19 has been shown to interact with various signaling molecules including β-catenin/RAC1[22], Egr1 [23], HER2 [24], and GSK3β [25] to regulate their subcellular localization. In this study, we explored the possibility of an interaction between K19 and HNRNPK and the indirect impact of K19 on posttranscriptional gene regulation of HNRNPK target mRNAs by changing the balance of nucleocytoplasmic HNRNPK localization. We found that the direct interaction between K19 and HNRNPK was required for cytoplasmic HNRNPK localization in the MDA-MB-231 breast cancer cells. By using photoactivatable ribonucleoside-enhanced crosslinking and immunoprecipitation (PAR-CLIP) to identify mRNAs bound to HNRNPK along with RNA-seq data, we identified genes whose abundance are affected by K19. The top pathways of genes regulated by K19 and cytoplasmic HNRNPK were related to cancer signaling and included the p53 tumor suppressor pathway. These findings reveal that K19 regulates HNRNPK functions in gene expression through its physical interaction, ultimately affecting gene expression in the mutant p53 signaling pathway. The oncogenic role of K19 was confirmed by assessing cell proliferation in the K19 context and finding that K19 is required for cell proliferation.

## Results

### K19 binds to HNRNPK and promotes its cytoplasmic localization

We set out to study the effect of K19 on HNRNPK localization and posttranscriptional gene regulatory activity. We used MDA-MB-231 triple-negative breast cancer (TNBC) cells with CRISPR-Cas9 mediated *KRT19* knockout (KO) [26] and confirmed complete ablation of K19 expression by Western blotting (Fig. S1A) and RNA-sequencing (RNA-seq) (Table S1).

**Fig. 1:**
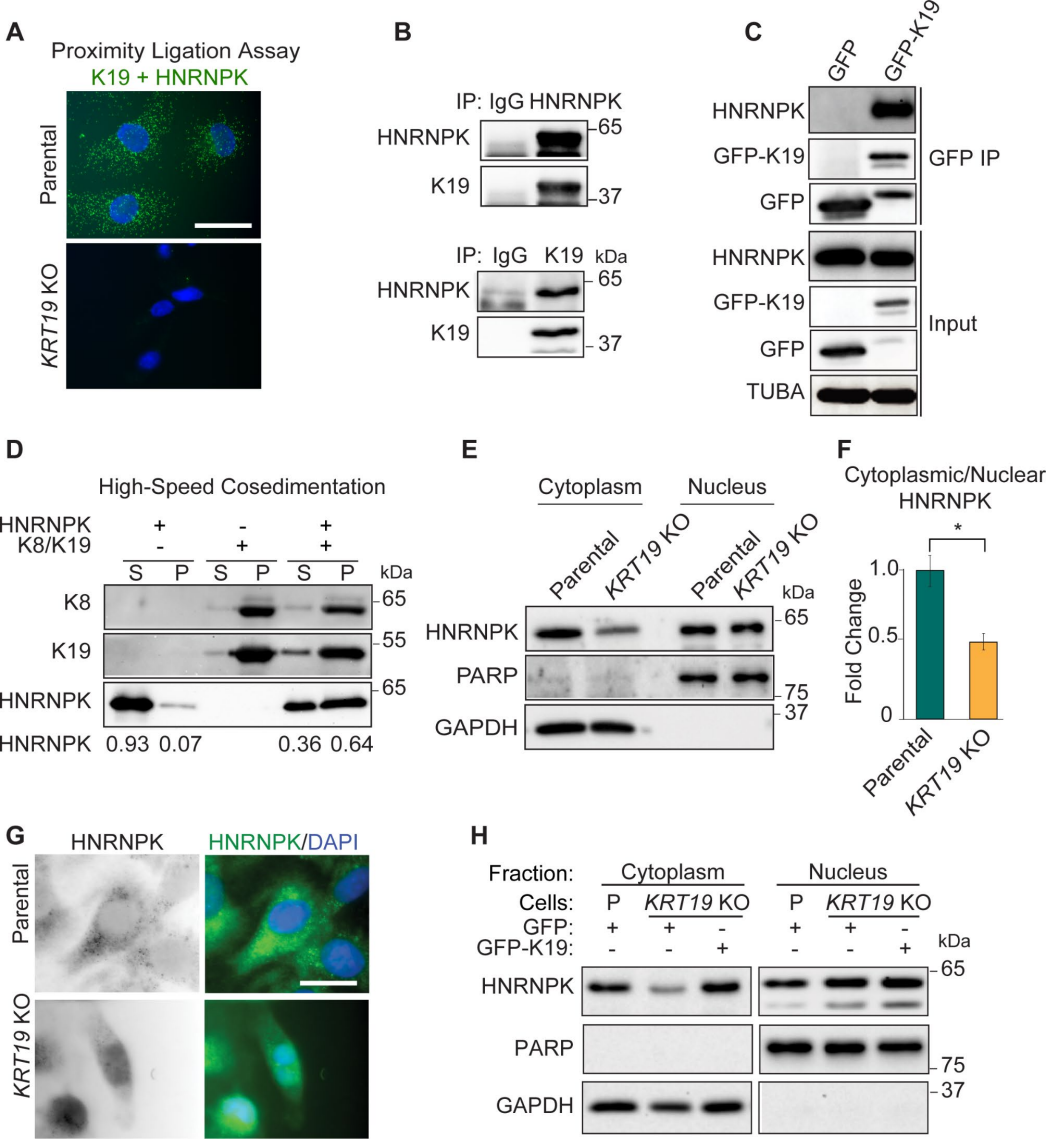
K19 binds to HNRNPK and promotes its cytoplasmic localization. **(A)** Proximity ligase assay showing K19 and HNRNPK colocalization in MDA-MB-231 cells. Bar = 10 μm. **(B)** Co-IP of K19 and HNRNPK using IgG as a control. **(C)** Co-IP of K19 and HNRNPK using GFP and GFP-19 tagged transiently expressing HEK293 cells. Full-length blots/gels are presented in Supplementary Fig. 7. **(D)** High-speed in vitro co-sedimentation assay following recombinant HNRNPK incubation with (+) or without (-) preassembled filaments of K19 and its obligate polymerization partner K8. Supernatant (S) and pellet (P) were separated, and immunoblotting was performed. **(E)** Biochemical subcellular fractionation showing decreased cytoplasmic HNRNPK in *KRT19* knockout cells. PARP was used as a loading control for nuclear fraction while GAPDH was used as loading control for cytoplasmic fraction. Full-length blots/gels are presented in Supplementary Fig. 8. **(F)** Quantification of cytoplasmic versus nuclear HNRNPK in MDA-MB-231 cells. **(G)** Immunofluorescence of cells (N = nucleus) permeabilized using mild detergent (bar = 10 μm). **(H)** Biochemical subcellular fractionation in both parental (P) and *KRT19* KO cells showing increased cytoplasmic HNRNPK in *KRT19* KO cells upon overexpression K19. Full-length blots/gels are presented in Supplementary Fig. 9

Keratins are an interaction platform for various proteins and a previous study showed that keratin closely related to K19, K17, interacts with HNRNPK in skin cancer cells [4]. To test a potential interaction between K19 and HNRNPK, we performed proximity ligation assays (PLA). PLA using K19 and HNRNPK antibodies after permeabilizing with a mild detergent (0.01% digitonin) showed cytoplasmic fluorescence signals from parental cells but not from *KRT19* KO cells (Fig. 1A). PLA using K19 and HNRNPK antibodies after permeabilizing with triton also showed that K19 interacts with HNRNPK (Fig. S1B). We further validated the K19-HNRNPK interaction by co-immunoprecipitation (co-IP) of endogenous proteins and found that K19 and HNRNPK were detected in IPs of HNRNPK and K19, respectively (Fig. 1B). Consistently, HNRNPK specifically co-immunoprecipitated with transiently expressed GFP-tagged K19 in HEK293 cells but not with the GFP control (Fig. 1C).

We then tested for a direct interaction between K19 filaments and HNRNPK in vitro by high-speed co-sedimentation. For this, filaments were preassembled using purified K19 and its oligomerization partner keratin 8 (K8). Next, recombinant HNRNPK was incubated with or without preassembled K8/K19 filaments. After high-speed centrifugation, sedimentation of recombinant HNRNPK increased significantly in the presence of K8/K19 filaments, whereas HNRNPK remained mostly in the supernatant fraction without filaments (Fig. 1D), indicating a direct interaction between HNRNPK and K19 filaments.

Keratin filaments regulate the subcellular localization of several proteins [25, 27–29], and we hypothesized that K19 may influence the localization of the typically predominantly nuclear HNRNPK. Therefore, we examined the cytoplasmic and nuclear levels of HNRNPK in parental and *KRT19* KO cells. Subcellular fractionation revealed a decrease in cytoplasmic HNRNPK level and cytoplasmic to the nuclear ratio of HNRNPK in *KRT19* KO cells (Fig. 1E, F) without changes in overall HNRNPK levels (Fig. S1A, C). Immunostaining of cytoplasmic HNRNPK after mild permeabilization also showed a higher HNRNPK signal from the cytoplasm of parental cells compared to *KRT19* KO cells (Fig. 1G). Finally, the rescue of cytoplasmic HNRNPK levels by stable expression of GFP-K19 in *KRT19* KO cells confirmed the requirement of K19 for cytoplasmic HNRNPK accumulation (Fig. 1H).

### K19 regulates cytoplasmic HNRNPK targets

**Fig. 2:**
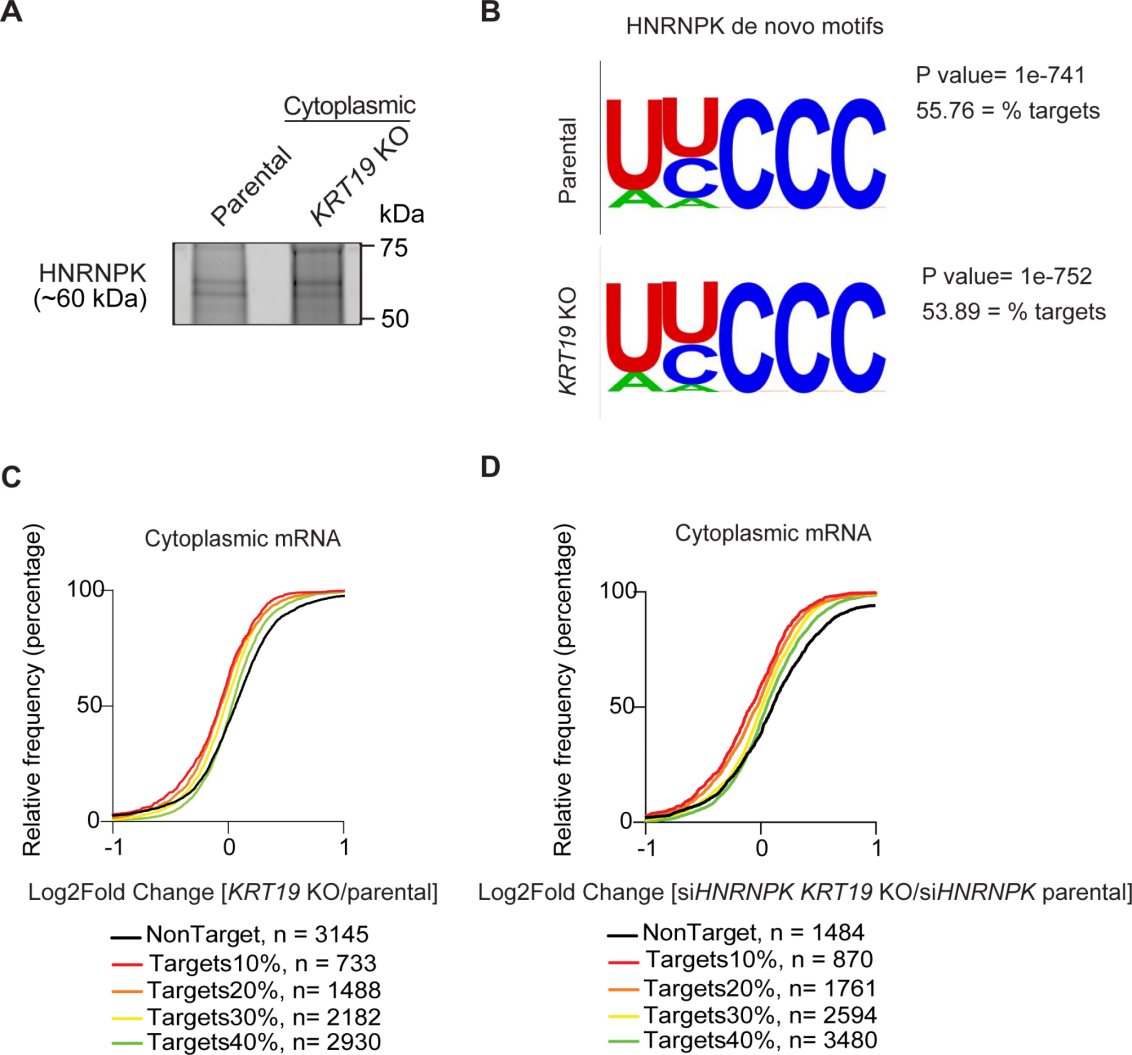
K19 regulates mRNAs directly bound to HNRNPK. **(A)** SDS-PAGE fluorescent imaging of the crosslinked cytoplasmic HNRNPK immunoprecipitation revealed two bands migrating at ~ 60 kDa, corresponding to HNRNPK (upper band) and HNRNPJ (lower band), a putative spliced isoform of HNRNPK, in both cell lines. **(B)** Sequence logo from cytoplasmic HNRNPK parental and *KRT19* KO cells. **(C)** The empirical cumulative percentages plot of the change in mRNA levels in *KRT19* KO compared to parental. The analysis compares HNRNPK targets (top 10%, Red, n = 733), (top 20%, Orange, n = 1488), (top 30%, Yellow, n = 2182), (top 40%, Green, n = 2930), and non-targets (Black, n = 3145). **(D)** The empirical cumulative percentages plot of the change in mRNA levels in cells transiently depleted HNRNPK in both parental and *KRT19* KO. The analysis compares HNRNPK targets (top 10%, Red, n = 870), (top 20%, Orange, n = 1761), (top 30%, Yellow, n = 2594), (top 40%, Green, n = 3480), and non-targets (Black, n = 1484). Targets (colored lines) binned by number of HNRNPK binding sites and non-targets (black line) with minimal gene expression of 4 fragments per kilobase of exon per million mapped fragments ((FPKM) ≥ 4) are shown

HNRNPK is expected to post-transcriptionally regulate its RNA targets in the cytoplasm [16] and therefore we examined whether loss of K19 and concomitant loss of cytoplasmic HNRNPK resulted in changes in HNRNPK target RNA levels. First, we comprehensively mapped the RNA interactome of cytoplasmic HNRNPK and characterized its RNA recognition elements (RREs) using 4-thiouridine (4SU) PAR-CLIP in both parental and *KRT19* KO cells (Fig. S2) [30]. In these cells, RNA and proteins were crosslinked by UV, then subcellular fractionation of both parental and *KRT19* KO cells was performed. HNRNPK from the cytoplasmic fraction was immunoprecipitated, and the RNA covalently attached in immunoprecipitated ribonucleoproteins (RNPs) was partially digested and fluorescently labeled. SDS-PAGE fluorescent imaging of the cytoplasmic HNRNPK immunoprecipitated revealed two bands migrating at ~ 60 kDa, corresponding to ribonucleoprotein complexes containing HNRNPK (upper band) and its putative spliced variant HNRNPJ (lower band) (Fig. 2A) [31, 32]. We recovered and sequenced cytoplasmic HNRNPK-bound RNA fragments from MDA-MB-231 parental and *KRT19* KO cells and used PARalyzer to identify clusters of overlapping reads harboring the characteristic T-to-C mutations indicating RNA-protein crosslinking events [33] (Tables S2, S3). Using HOMER, we found that our PAR-CLIP binding sites were highly enriched for C/U-rich sequences in both parental and *KRT19* KO, consistent with the previously reported HNRNPK RRE [13, 34] (Fig. 2B).

We next examined the role of K19 on the steady-state abundance of HNRNPK targets and integrated RNA-seq results from parental and *KRT19* KO cells (Table S1) with our PAR-CLIP data. We found that the abundance of cytoplasmic HNRNPK targets were reduced in *KRT19* KO cells, and the magnitude of this effect depended on the overall strength of HNRNPK binding as defined by the number of cytoplasmic HNRNPK binding sites (Fig. 2C). In particular, levels of mRNAs with HNRNPK-binding sites at their 3’ untranslated region (UTR), coding, exon, and introns were decreased in *KRT19* KO cells (Fig. S3A-D). In contrast, mRNAs with HNRNPK-binding sites at the 5’ UTRs region were not affected in *KRT19* KO cells (Fig. S3E).

The cytoplasmic HNRNPK pool likely protects target mRNAs by increasing their steady-state abundance through competition with other RBPs at 3’ UTR, analogous to the function of other RBPs [35]. Our findings suggest that Keratin 19 plays a role in posttranscriptional gene regulation by affecting subcellular localization of HNRNPK, thereby altering the expression of HNRNPK target mRNAs bound to HNRNPK via 3’ UTR.

To confirm the regulating role of K19 on HNRNPK-dependent transcripts, we knocked down HNRNPK using siRNAs in both parental and *KRT19* KO cells (Fig. S4). The abundance of HNRNPK targets in siHNRNPK-treated *KRT19* KO cells was reduced compared to those in siHNRNPK-treated parental cells (Fig. 2D), further indicating that K19 regulates HNRNPK-dependent transcripts. Additionally, cytoplasmic HNRNPK targets bound to 3’ untranslated region, coding, exon, and introns were downregulated in *KRT19* KO cells upon knocking down HNRNPK, meaning that both K19 and HNRNPK together are needed to maintain levels of cytoplasmic HNRNPK targets (Fig. S3F-J). Collectively, these results suggest that although K19 has little to no effect on the mechanism of nucleic acid recognition by HNRNPK, K19 indirectly affects the abundance of HNRNPK target mRNAs by controlling its cytoplasmic accumulation.

### K19 is required to maintain levels of cytoplasmic HNRNPK target mRNAs

**Fig. 3:**
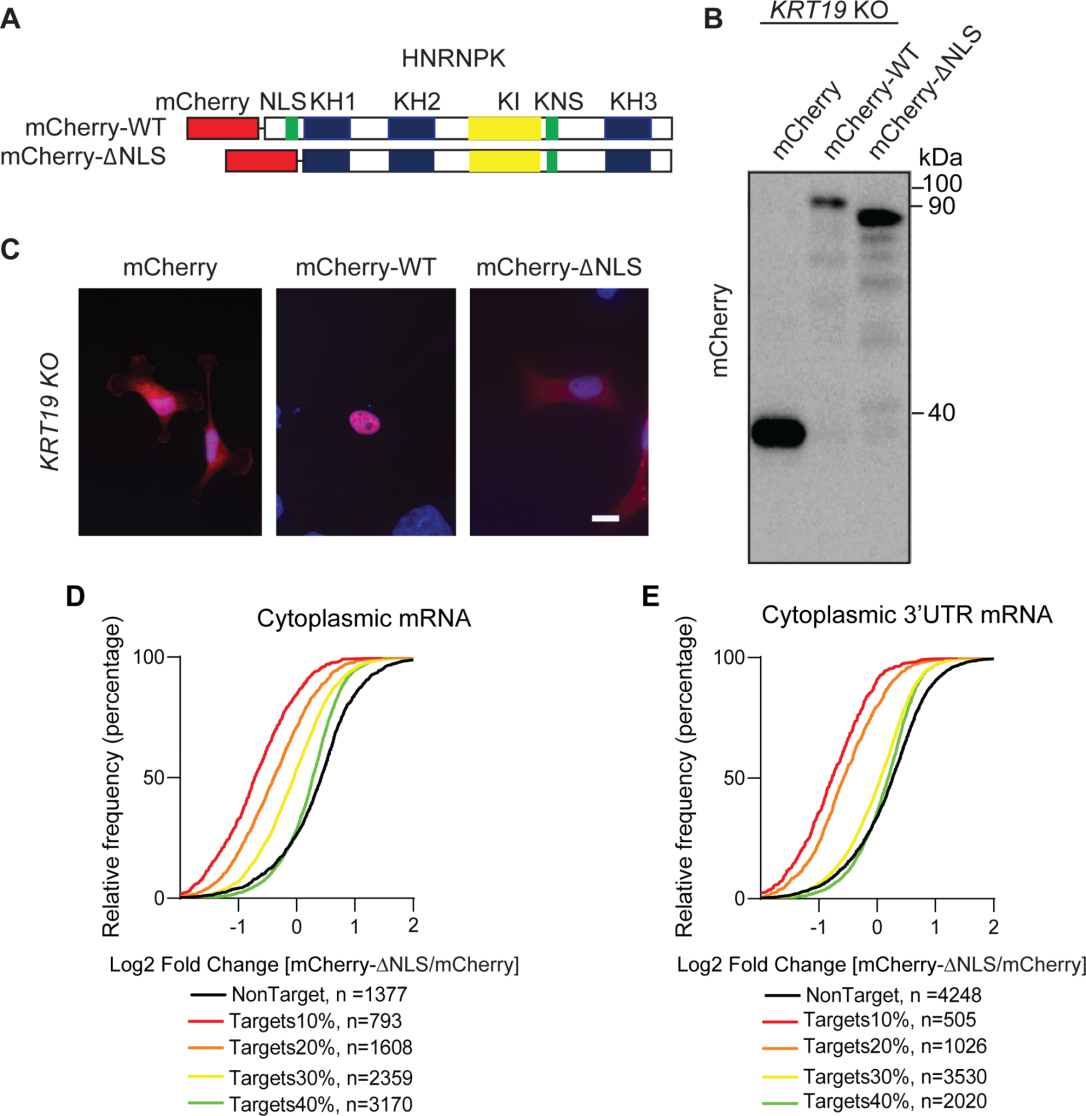
Overexpressing cytoplasmic HNRNPK decreases levels of cytoplasmic HNRNPK targets. **(A)** Schematics of wildtype (WT) HNRNPK and cytoplasmic mutant (ΔNLS). N-termini of WT and ΔNLS HNRNPK were tagged with mCherry (mCherry-WT and mCherry-ΔNLS, respectively). **(B)** Immunoblot from lysates of *KRT19* KO cells transfected with either mCherry, mCherry-WT, or mCherry-∆NLS using an antibody against mCherry. **(C)** *KRT19* KO cells transiently transfected with mCherry, mCherry-WT, or mCherry-∆NLS were immunostained with anti-RFP antibody. Nuclei are shown with DAPI (bar = 20 μm). The empirical cumulative distribution function of mRNA expression changes in MDA-MB-231 parental cells transiently transfected with mCherry or mCherry-∆NLS with genes binned by HNRNPK binding. **(D)** The empirical cumulative percentages plot of the change in cytoplasmic mRNA. The analysis compares HNRNPK targets (top 10%, Red, n = 793), (top 20%, Orange, n = 1608), (top 30%, Yellow, n = 2359), (top 40%, Green, n = 3170), and non-targets (Black, n = 1377). **(E)** The empirical cumulative percentages plot of the change in cytoplasmic 3’UTR mRNA, The analysis compares HNRNPK targets (top 10%, Red, n = 505), (top 20%, Orange, n = 1026), (top 30%, Yellow, n = 3530), (top 40%, Green, n = 2020), and non-targets (Black, n = 4248). Targets (colored lines) binned by number of HNRNPK binding sites and non-targets (black line) with minimal gene expression of 4 fragments per kilobase of exon per million mapped fragments ((FPKM) ≥ 4) are shown

We then examined whether overexpressing cytoplasmic HNRNPK can rescue the decreased levels of HNRNPK targets occurring in the absence of K19. To specifically overexpress cytoplasmic HNRNPK, we created an expression construct with a deleted nuclear localization signal (HNRNPK ∆NLS), which indeed localized HNRNPK to the cytoplasm (Fig. 3A-C). mCherry, which was used to tag HNRNPK ∆NLS, did not affect the K19-mediated stabilization of cytoplasmic HNRNPK targets (Fig. S5). Integrating the list of cytoplasmic HNRNPK targets (Table S2) with RNA-seq results from *KRT19* KO cells overexpressing HNRNPK ∆NLS or vector (Table S4) showed no change in levels of cytoplasmic HNRNPK targets (data not shown). However, integrating the list of cytoplasmic HNRNPK targets (Table S2) with RNA-seq results from parental cells overexpressing HNRNPK ∆NLS or vector (Table S5) revealed a decrease in abundance of HNRNPK target mRNAs when HNRNPK ∆NLS was overexpressed (Fig. 3D). This decrease was independent of whether the targets were binned by the overall number of HNRNPK binding sites or by the number of binding sites only in the target mRNA 3’ UTR (Fig. 3E). Altogether, these data suggest that the ability of the HNRNPK to stabilize its targets in the cytoplasm requires the presence of K19 as well as the ability of HNRNPK to enter the nucleus and transport targets from it.

### K19 and HNRNPK co-regulate p53 signaling pathway

**Fig. 4:**
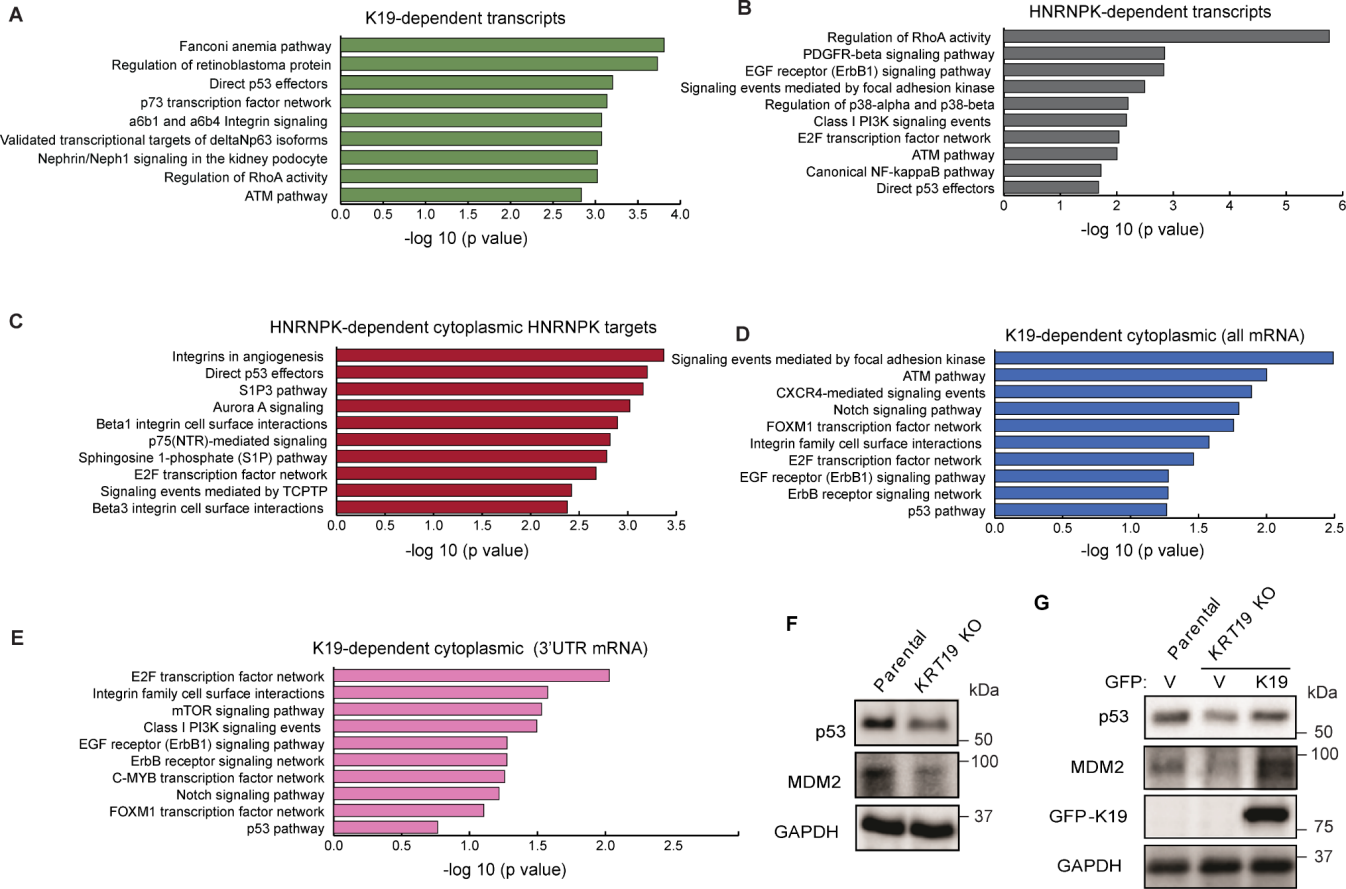
Gene ontology identifies that K19 and HNRNPK promote p53 signaling pathway in MDA-MB-231 cells. **(A)** The top 10 enriched NCI-Nature pathways for mRNAs downregulated by *KRT19* KO. The X axis denotes the number of genes while Y axis denotes NCI-Nature pathways terms. **(B)** The top 10 enriched NCI-Nature pathways for mRNAs downregulated by HNRNPK KD. The X axis denotes the number of genes while Y axis denotes NCI-Nature pathways terms. **(C)** The top 10 enriched NCI-Nature pathways for Cytoplasmic HNRNPK mRNAs downregulated PAR-CLIP targets after HNRNPK KD or (all mRNAs) upon overexpressing cytoplasmic HNRNPK. **(D)** The top 10 enriched NCI-Nature pathways for Cytoplasmic HNRNPK mRNAs downregulated PAR-CLIP targets after *KRT19* KO or (all mRNAs) upon overexpressing cytoplasmic HNRNPK. **(E)** Cytoplasmic HNRNPK (3’UTR mRNAs) upon overexpressing cytoplasmic HNRNPK. The X axis denotes the number of genes while the Y axis denotes NCI-Nature pathways terms. Enriched GO terms among downregulated HNRNPK GO terms for top 10 pathways based on p values are shown. **(F)** Immunoblots from lysates of Parental MDA-MB-231 and *KRT19* KO cells or **(G)** Parental and *KRT19* KO cells stably expressing GFP or GFP-K19 with antibodies against the indicated proteins. Full-length blots/gels are presented in Supplementary Figs. 10–11

We next identified molecular pathways regulated by HNRNPK that are dependent on K19 to find gene networks that may give insight into the apparent association of K19 loss with cell proliferation. First, we analyzed gene ontology (GO) pathways for genes that were K19-dependent or downregulated in *KRT19* KO cells (Fig. 4A). Signaling pathways including the Fanconi anemia pathway, regulation of retinoblastoma protein, and direct p53 effectors were significantly affected in the absence of K19. Similarly, signaling pathways involving genes downregulated in HNRNPK KD (Table S6) included the E2F transcription factor network, Aurora A signaling, Integrin in angiogenesis, and direct p53 effectors (Fig. 4B). Further GO analysis performed using cytoplasmic HNRNPK mRNAs downregulated PAR-CLIP targets after HNRNPK KD in parental cells (Table S7) showed direct p53 effectors remained as one of the top enriched pathways (Fig. 4C). We also performed the same analysis upon overexpressing cytoplasmic HNRNPK in *KRT19* KO cells, examining HNRNPK mRNAs via PAR-CLIP for all mRNAs (Fig. 4D) and 3’ UTR targets (Fig. 4E, Table S8) and found that pathways involving p53 were still among top 10 affected pathways.

To confirm these observations from GO analyses, we decided to examine levels of p53 whose mRNA was a K19- and HNRNPK-dependent cytoplasmic HNRNPK target (Tables S6, S7). The dysregulation of the p53 pathway by loss of expression or mutation of its components is one of the major drivers promoting cancer cell survival and metastasis and the MDA-MB-231 cell line expresses high levels of the p53 R280K mutant [36]. A link between HNRNPK and p53 was previously established by reports documenting that HNRNPK serves as a p53 partner and regulator in response to stress [12, 37]. We decided to test whether K19 loss changes expression levels of p53 or its activator MDM2. We found that p53 and MDM2 levels are decreased in *KRT19* KO cells (Fig. 4F). This decrease was dependent on K19, as the reintroduction of GFP-K19 rescued p53 and MDM2 expression levels (Fig. 4G). Altered p53 pathway gene expression may thus contribute to the observed decreased cancer cell proliferation accompanying K19 loss.

### K19 knockout reduces proliferation of MDA-MB-231 cells

**Fig. 5:**
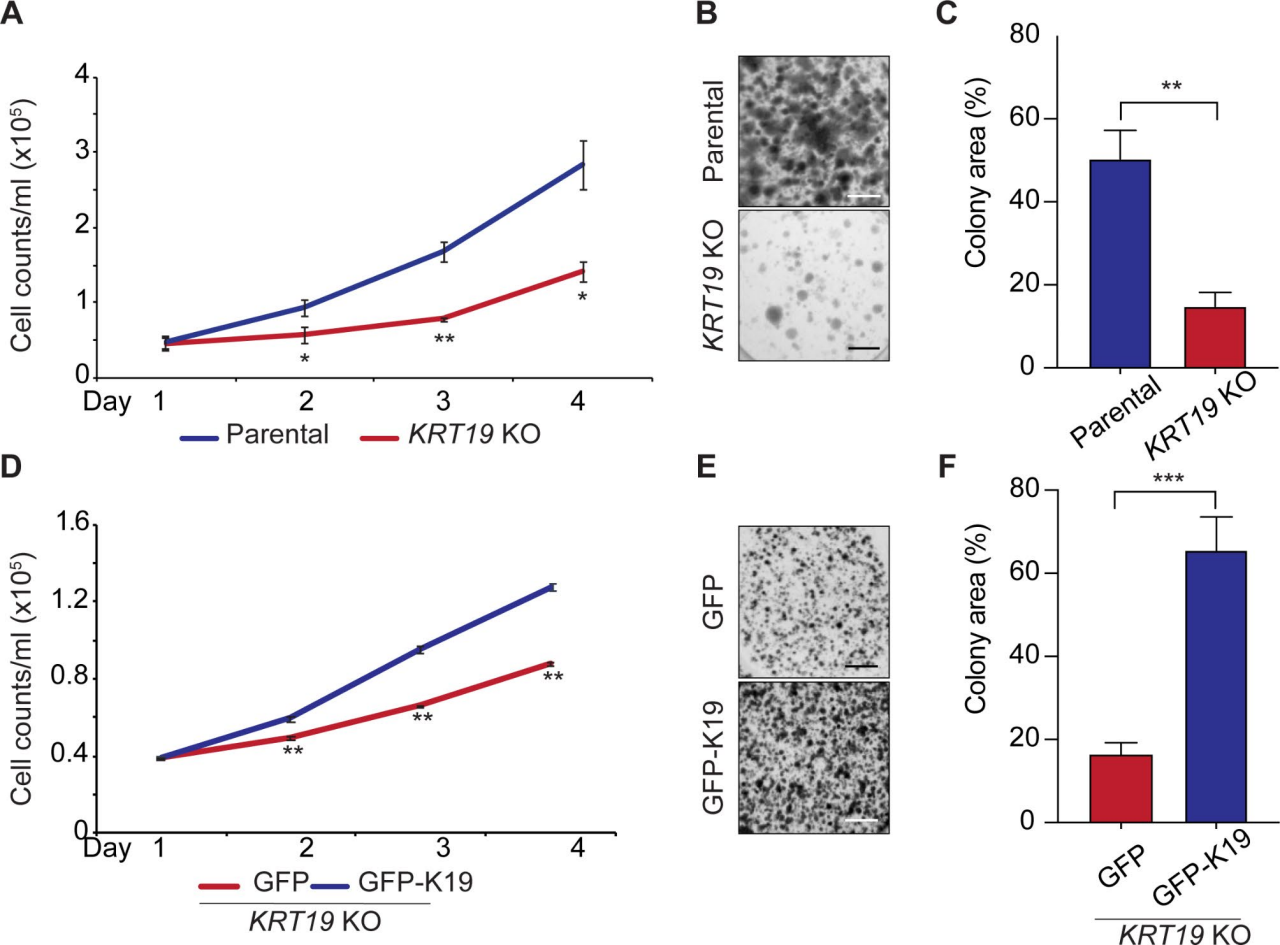
Cell proliferation of MDA-MB-231 breast cancer cells requires K19. **(A)** After 50,000 cells were plated on Day 0, cell numbers were counted every 24 h, up to 96 h, using a hemocytometer. **(B)** 1000 cells were plated on each well of 6 well plates. After growing cells for two weeks, crystal violet staining was performed to visualize colonies, and images of plates were taken. **(C)** The colony area was quantified using the ImageJ software. **(D)** After 50,000 *KRT19* KO cells transfected with either GFP or GFP-K19 were plate on Day 0, cell numbers were counted every 24 h, up to 96 h, using a hemocytometer. **(E)** 10,000 cells were plated on each well of 6 well plates. After growing cells for two weeks, crystal violet staining was performed to visualize colonies, and images of plates were taken. **(F)** The colony area was quantified using the ImageJ software. Data from at least three technical are shown as mean ± SEM. *** p < 0. 001. Scale bar 0.5 mm

Since mutant p53 was already known to enhance cancer cell proliferation through binding to other effector proteins like cyclins, we investigated this role after manipulating K19 levels. Previous reports have shown that loss of K19 in estrogen receptor-positive MCF7 breast cancer cells resulted in a delayed cell cycle [21]. We tested whether K19 also affected the proliferation of MDA-MB-231 cells by cell counting and found that *KRT19* KO cells showed significantly decreased cell proliferation compared to the parental control (Fig. 5A). Similarly, *KRT19* KO cells showed significantly reduced cell proliferation in a colony formation assay (Fig. 5B, C). We confirmed the specificity of the *KRT19* KO effect on cell proliferation by rescuing K19 expression with a GFP-K19 construct (Fig. S6) in our cells, which as expected, resulted in increased cell proliferation by cell counting (Fig. 5D) and colony formation assays (Fig. 5E, F). Taken together, our findings show that K19 promotes MDA-MB-231 cell proliferation.

## Discussion

Overall, we showed that HNRNPK and K19 physically interact in the cytoplasm, and K19 and HNRNPK co-regulate the p53 signaling pathway. We propose that K19 filaments serve as cytoplasmic loading docks for HNRNPK.

Since HNRNPK is a nucleocytoplasmic shuttling protein [18], the interaction between K19 and HNRNPK in the cytoplasm and HNRNPK co-sedimentation with K8/K19 filaments indicate that HNRNPK becomes associated with K8/K19 filaments in the cytoplasm after exiting the nucleus. Since higher levels of K19 and cytoplasmic localization of HNRNPK in tumors have been correlated with poor patient survival rate and metastasis [8, 38, 39], our data demonstrate a potential explanation of these clinical observations on patient survival.

Interestingly, we found that levels of mRNAs with HNRNPK-binding sites in introns were higher in the presence of K19, supporting the possibility that HNRNPK generally binds to all its targets first in the nucleus and then shuttles as part of a larger RNP into the cytoplasm. In this regard, keratins have been found inside the nucleus, where they interact with nuclear proteins [40], and K19 contains a bipartite nuclear localization signal towards its C-terminus [40]. Due to its role in RNA transport and maturation [41, 42], HNRNPK likely emerges from the nucleus already bound to its targets. In this scenario, the interaction with K19 would allow HNRNPK to remain bound to and stabilize its targets better. Preloading of HNRNPK to its preferred targets may also explain why the overall number of PAR-CLIP binding sites was predictive of HNRNPK-mediated regulation of target RNA abundance in the cytoplasm, even if they were not exclusively found in the cytoplasm, but also introns.

Using our *KRT19* KO system, we identified a comprehensive list of HNRNPK-dependent mRNAs in the cytoplasm. The list includes the E2F transcription factor network, which we reported to be K19-dependent using hormone-positive breast cancer cells [21]. Identification of genes involved in promoting cancer and cell migration is a strong indication that transcriptomic changes of those genes significantly contribute towards K19-dependent cell proliferation and cytoplasmic HNRNPK-dependent cancer metastasis. Therefore, we have not only determined how HNRNPK localizes to the cytoplasm but have also identified novel cytoplasmic RNA targets of HNRNPK in breast cancer cells.

Given the fact that both K19 and HNRNPK are members of large families of proteins, it is likely that there are other intermediate filament protein-HNRNP interactions. Indeed, K17 interacts with HNRNPK as mentioned above, and HNRNPS1 proteins have been shown to interact with a type III intermediate filament protein vimentin, which is upregulated in several cancers and plays a critical role in metastasis [43]. Therefore, our findings can be used to characterize the mechanism underlying the progression of various tumor types.

## Experimental procedure

### Cell culture

The MDA-MB-231 cell line was a generous gift from Dr. Zaver Bhujwalla (Johns Hopkins School of Medicine, Baltimore, MD). MDA-MB-231 cells were authenticated by short-tandem repeat profiling, performed by ATCC. MDA-MB-231 *KRT19* KO cells generated using the CRISPR/Cas9 system and cells stably expressing GFP or GFP-*KRT19* generated using the lentiviral system were described previously [26]. Stable transductants were selected using hygromycin (100 µg/ml). Cells were grown in Dulbecco’s modified essential medium (DMEM; Gibco, Grand Island, NY) supplemented with 10% Fetal Bovine Serum (GE Healthcare, Logan, UT) and 1% penicillin/streptomycin (Gibco) in a humidified incubator at 5% CO_2_ and 37 °C. To measure cell proliferation, 50,000 cells were plated on each well of six-well plates. Cells were counted using a hemocytometer 24, 48, 72, and 96 h after plating.

### Plasmids and siRNAs

To overexpress HNRNPK, cDNA was cloned out of pCMV6-AC *HNRNPK* (OriGene, Rockville, MD) using the primers described previously [4] and cloned into pmCherry-C1 (Takara Bio Inc., San Jose, CA). To silence HNRNPK expression, Accell Human SMARTpool *HNRNPK* siRNAs (Dharmacon, Lafayette, CO) was used. The siRNA sequences targeting *HNRNPK* are described in Table 1. AllStars negative control SI03650318 was purchased from Qiagen (Germantown, MD).

**Table 1:** Sequences for Accell Human SMARTpool *HNRNPK* siRNAs from Dharmacon

Gene	siRNA sequence
*HNRNPK* #1	5’-GUAGAGUGCAUAAAGAUCA-3’
*HNRNPK* #2	5’-CCUAUGAUUAUGGUGGUUU-3’
*HNRNPK* #3	5’-GCCUUAUGAUCCCAAUUUU-3’
*HNRNPK* #4	5’-GCAUUUGUAUUGAUAGUUA-3’

### Transient transfection

For siRNA, RNAimax lipofectamine (Invitrogen, Waltham, MA) was used to transfect *HNRNPK* smartpool siRNA (Dharmacon) or non-targeting siRNA (Qiagen) according to the manufacturer’s protocol. For overexpression plasmids, jetOptimus DNA transfection reagent (Polyplus, Illkirch, France) was used according to the manufacturer’s protocol.

### PAR-CLIP analysis

Both parental and *KRT19* KO cells were treated with 100 µM 4thiouridine (4SU) for 16 h, washed with PBS and irradiated with 0.15 mJ cm^2^, 365 nm ultraviolet light in a Spectrolinker XL-1500 UV crosslinker to crosslink RNA to HNRNPK. Cytoplasmic pools of cells were collected by lysing them with a buffer containing 20 mM 4-(2-hydroxyethyl)-1-piperazineethanesulfonic acid (HEPES), pH 8, 1 mM ethylenediaminetetraacetic acid (EDTA), 1.5 mM magnesium chloride, 10 mM potassium chloride, 1 mM dithiothreitol, 1 mM sodium orthovanadate, 1 mM sodium fluoride, 1 mM phenylmethylsulfonyl fluoride, 0.5 mg/mL benzamidine, 0.1 mg/ml leupeptin, and 1.2 mg/mL aprotinin on ice for 15 min. Then, 7.5 µL of 10% NP-40 detergent was added to the cells and lysates were centrifuged for 1 min at 14000 g and 4°C, and supernatants were collected as cytosolic fractions. 10 U/ul of RNAse T1 was then added to the lysates followed by the immunoprecipitation using anti-HNRNPK antibody and Protein G magnetic beads. The beads were resuspended in one bead volume of dephosphorylation buffer after washing them with lysis buffer. On beads 3’ adapter ligation was performed, followed by on beads phosphorylation step. Then, the protein-RNA complexes were resolved using SDS-PAGE and fluorescent protein-RNA bands were recovered at the corresponding size ~ 60 kDa. Proteinase K digestion was performed followed by RNA recovery by acid phenol/chloroform extraction and ethanol precipitation. After RNA isolation, cDNA library preparation was carried out [30]. Library preparation was done using cDNA and sequences by Illumina platform. Obtained reads were processed and referenced to the hg19 genome assembly (hg19). Data analysis was performed by using PARalyzer settings where T-to-C mutation sequences were filtered. Data from the sequencer were converted to fastq files by being demultiplexed using Bcl2fastq (v2.20.0). Adapter barcoded samples were demultiplexed by using cutadapt (1.15 with python 3.6.4) with retaining the adapters. Also, libraries were demultiplexed by using 5’ adapter barcode by removing the 5’ adapter barcode anchored to 5’ end of the read. Moreover, data were further processed by PARpipe (https://github.com/ohlerlab/PARpipe) through implementing Paralyzer as well as annotation of groups and clusters. Potential PCR duplicates were further removed by allowing the use of additional UMIs in the new protocol and preserve the true number of reads in the final bam file, during preprocessing fastq sequences were collapsed prior to adapter and UMI removal [33, 44].

### RNA-seq analysis

For RNA-seq analysis, total RNAs from three biological replicates each of untransfected parental and *KRT19* KO cells, transfected with siHNRNPK, control siRNA, HNRNPK ∆NLS or vector were extracted. With siHNRNPK, control siRNA was extracted, while with HNRNPK ∆NLS, vector was extracted. Ribosomal RNA was depleted of using the NEBNext® rRNA Depletion Kit and cDNA libraries were prepared using the NEBNext® Ultra™ Directional RNA Library Prep Kit for Illumina® (NEB, Ipswich MA). RNA was barcoded using the NEBNext Multiplex Oligos for Illumina (NEB). All samples were multiplexed and sequenced on the Illumina HiSeq 3000 platform using 50 cycles single-end sequencing. Reads were aligned to hg19 using TopHat2 [45]. Cufflinks and Cuffdiff were used to quantify transcripts and determine differential expression. The top 500 upregulated genes with minimum 4 RPKM and p-value 0.05 were subjected to Enrichr tool, which uses curated gene sets to give biological meaning that can be used for further validation [2].

### Antibodies and other reagents

The following antibodies were used in this study anti-GAPDH (FL-335), anti-K19 (A-3), anti-K8 (C51), anti-K18 (C-04), anti-PARP (F-2), anti-HNRNPK (3C2) and anti-MDM2 (SMP14) were from Santa Cruz Biotechnology (Santa Cruz, CA); anti-p53 (DO-2) was from (MilliporeSigma, St. Louis, MO, USA); anti-GFP (12A6) was from the Developmental Studies Hybridoma Bank (Iowa City, IA); and anti-RFP (5F8) from ChromoTek (Munich, Germany).

### Western blotting

Cells were washed with 1X PBS, and cell lysates were prepared in cold Triton lysis buffer (1% Triton X-100; 40 mm HEPES, pH 7.5; 120 mm sodium chloride; 1 mm EDTA; 1 mm phenylmethylsulfonyl fluoride; 10 mm sodium pyrophosphate; 1 µg/ml each of chymostatin, leupeptin, and pepstatin; 10 µg/ml each of aprotinin and benzamidine; 2 µg/ml antipain; 1 mm sodium orthovanadate; and 50 mm sodium fluoride). Cell lysates were centrifuged to remove cell debris. Protein concentration was determined using the Bio-Rad Protein Assay (Bio-Rad) with bovine serum albumin as standard then was prepared in Laemmli SDS-PAGE sample buffer. Aliquots of protein lysate were resolved by SDS-PAGE, transferred to nitrocellulose membranes (Bio-Rad), cut into smaller pieces when necessary, and immunoblotted with the indicated antibodies, followed by horseradish peroxidase-conjugated goat anti-mouse or goat anti-rabbit IgG (MilliporeSigma, St. Louis, MO, USA) and Amersham ECL Select Western Blotting Detection Reagent or Pierce ECL Western Blotting Substrate (Thermo Scientific, Hudson, NH). Signals were detected using ChemiDoc Touch Imager (Bio-Rad) or CL1500 Imaging System (Thermo Fisher Scientific). For Western blot signal quantitation, the Image Lab software (Bio-Rad) was used.

### Biochemical subcellular fractionation

Subcellular fractionation was performed as described previously [4]. After rinsing with cold 1X PBS, cold lysis buffer (20 mM HEPES pH 8,1 mM EDTA, 1.5 mM Magnesium chloride, 10 mM Potassium chloride, 1 mM Dithiothreitol, 1 mM sodium orthovanadate, 1 mM Sodium fluoride, 1 mM phenylmethylsulfonyl fluoride, 0.5 mg/mL benzamidine, 0.1 mg/ml leupeptin, and 1.2 mg/mL aprotinin) was used to lyse the cells. Then 7.5 µL of 10% NP-40 detergent was added to the cells after incubating them on ice for 15 min. After adding NP-40 detergent to the cells, lysates were centrifuged for 1 min at 14,000 *g* and 4 °C, and supernatants were collected as cytosolic fractions. Pellets were washed 4 times with 1X PBS and incubated for 40 min at 4 °C (1% Triton X-100, 40 mM HEPES (pH 7.5), 120 mM sodium chloride, 1 mM EDTA, 1 mM phenylmethylsulfonyl fluoride, 10 mM sodium pyrophosphate, 1 µg/ml each of chymostatin, leupeptin and pepstatin, 10 µg/ml each of aprotinin and benzamidine, 2 µg/ml antipain, 1 mM sodium orthovanadate, 50 mM sodium fluoride). Supernatants were collected as nuclear fractions after centrifugation for 10 min at 13,800 *g* and 4 °C.

### Co-immunoprecipitation

Cells were washed with 1X PBS and cell lysates prepared in cold triton lysis buffer (1% Triton X-100; 40 mm HEPES (pH 7.5); 120 mm sodium chloride; 1 mm EDTA; 1 mm phenylmethylsulfonyl fluoride; 10 mm sodium pyrophosphate; 1 µg/ml each of chymostatin, leupeptin, and pepstatin; 10 µg/ml each of aprotinin and benzamidine; 2 µg/ml antipain; 1 mm sodium orthovanadate; 50 mm sodium fluoride) supplemented with 2% empigen for anti-K19 IP, or cold NP-40 lysis buffer (0.25% NP-40; 50 mM Tris (pH 8.0); 100 mM sodium chloride; 1 mm phenylmethylsulfonyl fluoride; 10 mm sodium pyrophosphate; 1 µg/ml each of chymostatin, leupeptin, and pepstatin; 10 µg/ml each of aprotinin and benzamidine; 2 µg/ml antipain; 1 mm sodium orthovanadate; 50 mm sodium fluoride) for anti-HNRNPK IP. Cell lysates were centrifuged to remove cell debris, and protein concentration was determined using the Bio-Rad Protein Assay with BSA as standard. Aliquots of cell lysate were then incubated with the indicated antibody or IgG control, and immune complexes were captured using Protein G Sepharose (GE Healthcare).

### Immunofluorescence (IF) staining

IF staining of cells was performed as described previously [26]. Cells grown on glass coverslips (VWR, Radnor, PA) were washed with 1X PBS, fixed in 4% paraformaldehyde in 1X PBS for 35 min, and permeabilized in 0.1% Triton X-100 for 20 min or 0.01% digitonin for 5 min. Samples were blocked in 5% normal goat serum (NGS; RMBIO, Missoula, MT) in 1X PBS before staining with primary antibodies diluted at 1:400 ratio in 5% NGS blocking buffer and a mixture of 1:1000 of Alexa Fluor 488-conjugated goat anti-mouse secondary antibody (Invitrogen) and 1:5000 DAPI (MilliporeSigma, St. Louis, MO, USA) in 1X PBS was added for 1 h incubation at RT. After 1X PBS washes, coverslips were mounted on microscope slides with a mounting medium containing 1,4-diaza-bicyclo[2.2.2]octane (Electron Microscopy Sciences, Hatfield, PA). Fluorescence images were taken using the Olympus optical elements fluorescence microscope (Olympus Optical Co., Japan).

### Proximity ligation assay

The Duolink in situ proximity ligation assay (PLA) was performed according to the manufacturer’s protocol (MilliporeSigma, St. Louis, MO, USA). In brief, cells were plated on glass coverslips, rinsed three times with PBS and fixed in 3.7% formaldehyde in 1X PBS for 20 min. The cells were permeabilized in 0.01% digitonin for 5 min and blocked with 5% NGS in 1X PBS for overnight at 4 °C. After blocking, cells were then incubated with antibodies against HNRNPK, K19 and IgG in 1X PBS containing 5% NGS overnight at 4 °C, followed by incubation with corresponding secondary antibodies conjugated with PLA probes for 60 min at 37 °C in the dark. Cells were washed three times in 1X PBS. Duolink and DAPI signals were detected using Olympus optical elements fluorescence microscope (Olympus). Images are analyzed using ImageJ [46].

### Colony formation assay

Colony formation assay was performed as described previously [25]. On each well of six-well plates, 1,000 cells were seeded and grown in 2 ml DMEM media for 14 days or 10,000 cells were seeded and grown for 10 days. The colonies were fixed with 4% formaldehyde and then stained with 0.5% crystal violet. Images were taken using ChemiDoc Touch Imager (Bio-Rad, Hercules, CA) and area of colonies were determined by ImageJ software (National Institutes of Health). Three biological replicates were analyzed.

### Graphs and statistics

All graphs in the manuscript are shown as mean ± standard error of mean. For comparisons between two datasets, a Student’s t test (tails = 2, type = 1) was used, and statistically significant p-values ≤ 0.05 are indicated in the figures and figure legends. GraphPad prism and excel were used to generate graphs in this manuscript.

### Electronic supplementary material

Below is the link to the electronic supplementary material.

Supplementary Material 1

Supplementary Material 2

Supplementary Material 3

Supplementary Material 4

Supplementary Material 5

Supplementary Material 6

Supplementary Material 7

Supplementary Material 8

Supplementary Material 9

## Data Availability

All data generated or analyzed during this study are included in this published article, its supplementary information files, and the GEO repository, GSE223603.

## Abbreviations

HNRNPK: Heterogeneous nuclear ribonucleoprotein
K19: Keratin 19
C-rich: Cytidine-rich
3’UTR: 3’ untranslated region
5’UTR: 5’ untranslated region
HNRNPK KD: HNRNPK knockdown
*KRT19* KO: keratin 19 knockout
KH: K-homology
KI: K interactive
CLIP: crosslinking and immunoprecipitation
K17: Keratin 17
PAR-CLIP: photoactivatable ribonucleoside-enhanced crosslinking and immunoprecipitation
TNBC: triple negative breast cancer
RNA-seq: RNA-sequencing
PLA: proximity ligation assays
co-IP: co-immunoprecipitation
K8: Keratin 8
RREs: RNA recognition elements
4SU: 4-thiouridine
RNPs: ribonucleoproteins
∆NLS: deleted nuclear localization signal
GO: gene ontology

## Declarations

### Disclaimer

This work was prepared while Byung Min Chung was employed at The Catholic University of America. The opinions expressed in this article are the author’s own and do not reflect the view of the National Institutes of Health, the Department of Health and Human Services, or the United States government.
