# Supplementary material for: Keratin 19 binds and regulates cytoplasmic HNRNPK mRNA targets in triple-negative breast cancer
Source: BMC Mol Cell Biol. 2023 Aug 17;24:26. doi: 10.1186/s12860-023-00488-z (PMC10433649; doi:10.1186/s12860-023-00488-z)
Supplement: Supplementary file 9 — Supplementary Material 9 [file 12860_2023_488_MOESM9_ESM.docx]

Keratin 19 binds and regulates cytoplasmic HNRNPK mRNA targets in triple negative breast cancer

Arwa Fallatah, Dimitrios G. Anastasakis, Amirhossein Manzourolajdad, Pooja Sharma, Xiantao Wang, Alexis Jacob, Sarah Alsharif, Ahmed Elgerbi, Pierre A. Coulombe, Markus Hafner and Byung Min Chung

**Supplementary Figures**


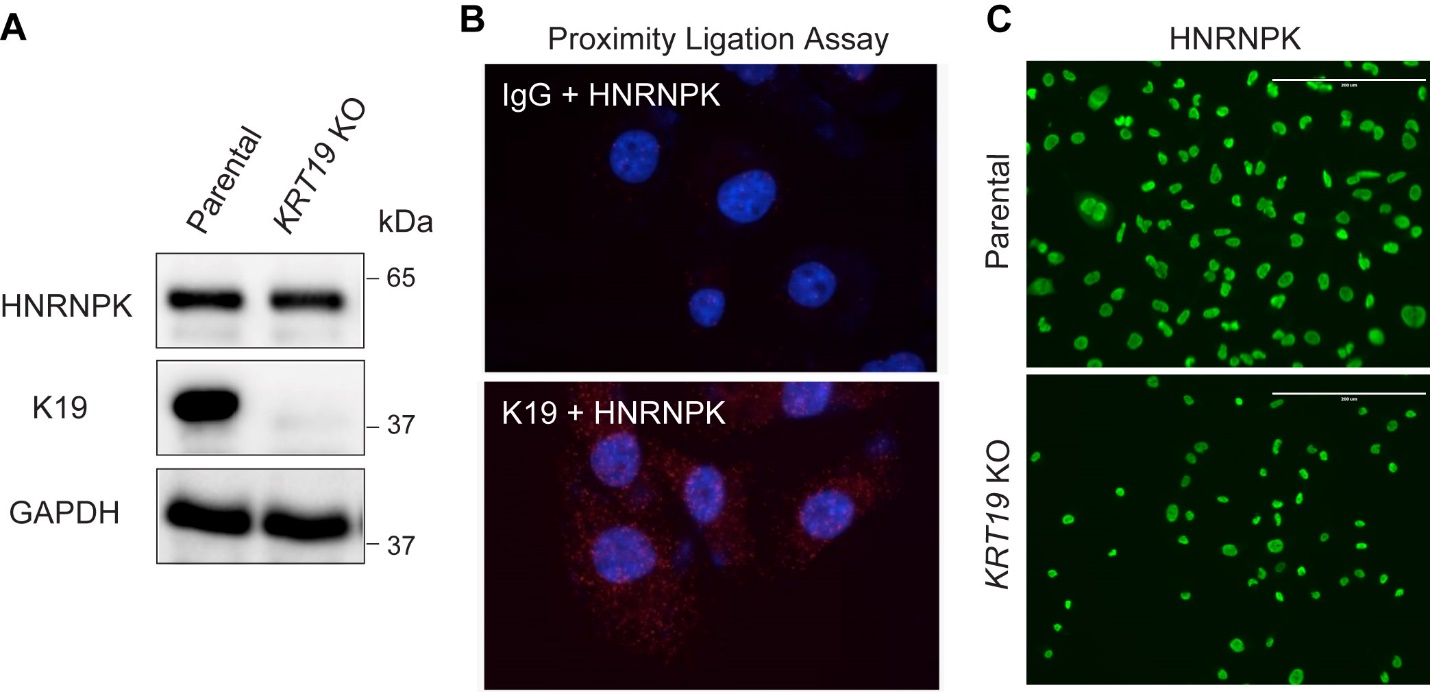


**Supplementary Figure 1.** K19-HNRNPK interaction and HNRNPK levels in KO cell lines. **A)** Whole cell lysates of parental control and *KRT19* KO cell lines were harvested, and immunoblotting was performed with antibodies against the indicated proteins. **B)** Proximity ligase assay (PLA) was performed using the indicated antibodies in MDA-MB-231 cells after the triton permeabilization. PLA signals are shown in red and DAPI is in blue. **C)** Parental and *KRT19* KO cells immunostained with anti-HNRNPK antibody (bar = 200 µm).


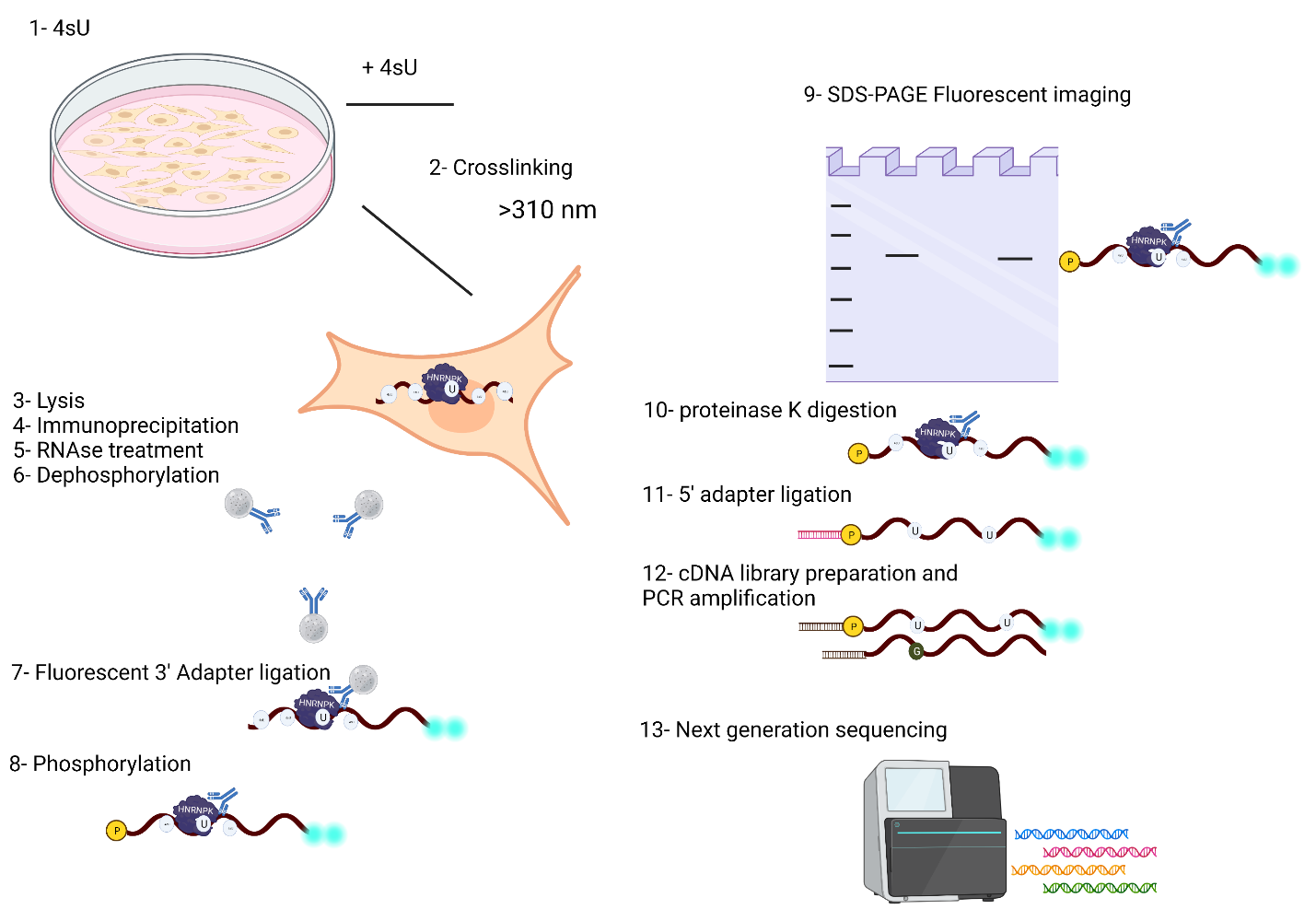


**Supplementary Figure 2. Flourescence-based PAR-CLIP schematic.** Cultured cells were treated with ribonucleoside analog 4sU, then crosslinked using UV radiation to incorporate T-to-C mutation to nascent RNA. Immunoprecipitation was then performed, and proteins were removed by digestion. Adaptors were ligated at both 3’ & 5’ ends and the samples were followed up with library preparation for next-generation sequencing.


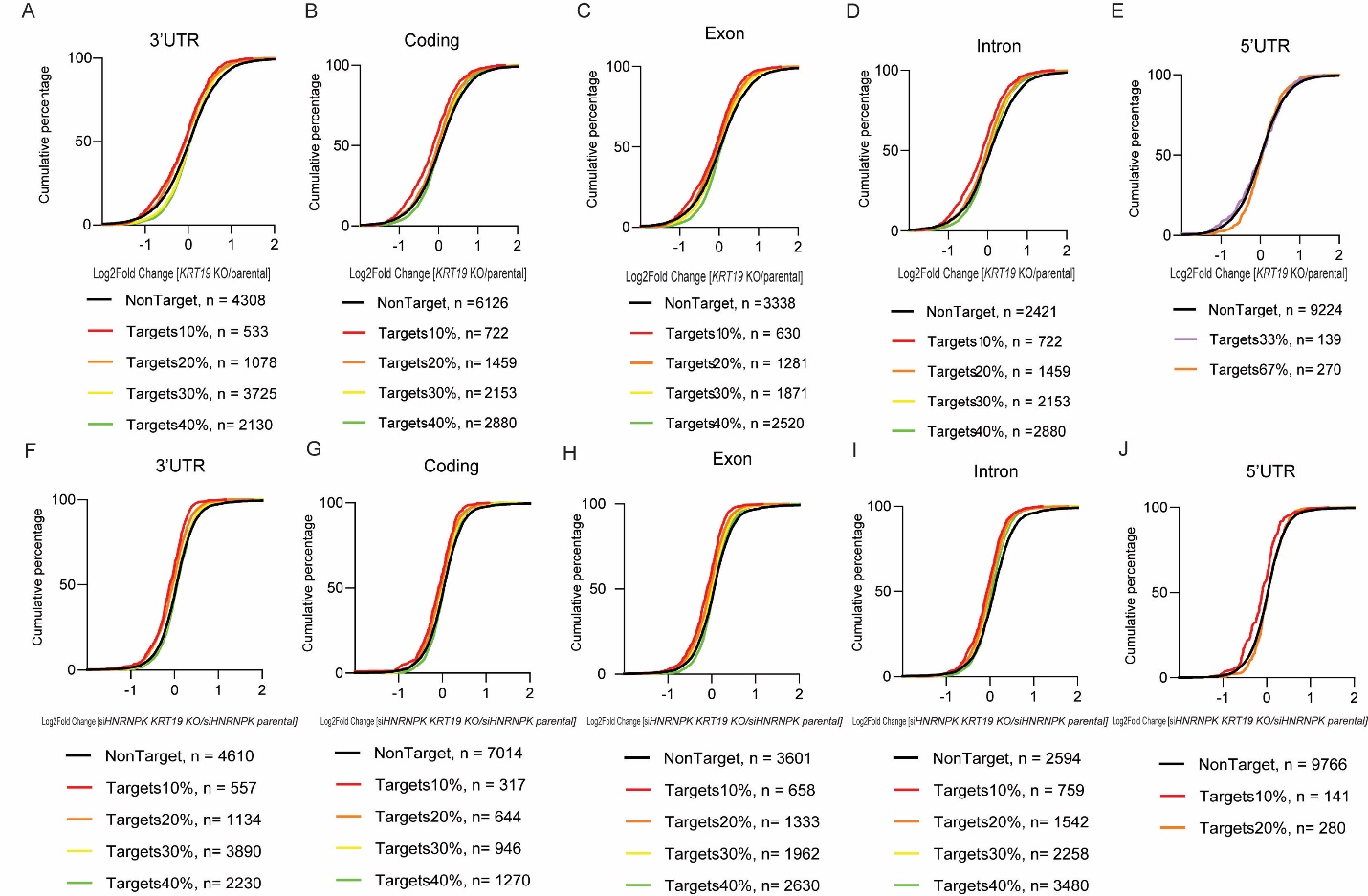


**Supplementary Figure 3.** K19 specifically destablized mRNAs bound to HNRNPK via 3’UTR, Coding, Exon, and Introns,3’. The empirical cumulative percentages of mRNA expression changes upon *KRT19* KO. mRNAs bound to HNRNPK were binned by top targets of **A)** 3’UTR, **B)** Coding, **C)** Exon, **D)** Intron, and **E)** 5’UTR region in *KRT19* KO and parental cells. The empirical cumulative percentages plot of the change in mRNA levels in *KRT19* KO compared to parental. **F)** 3’UTR, **G)** Coding, **H)** Exon, **I)** Intron, and **J)** 5’UTR region in *KRT19* KO and parental cells upon HNRPK KD. The empirical cumulative percentages plot of the change in mRNA levels in *KRT19* KO compared to parental. The analysis compares HNRNPK targets (top 10%, Re), (top 20%, Orange, (top 30%, Yellow), (top 40%, Green), and non-targets (Black).
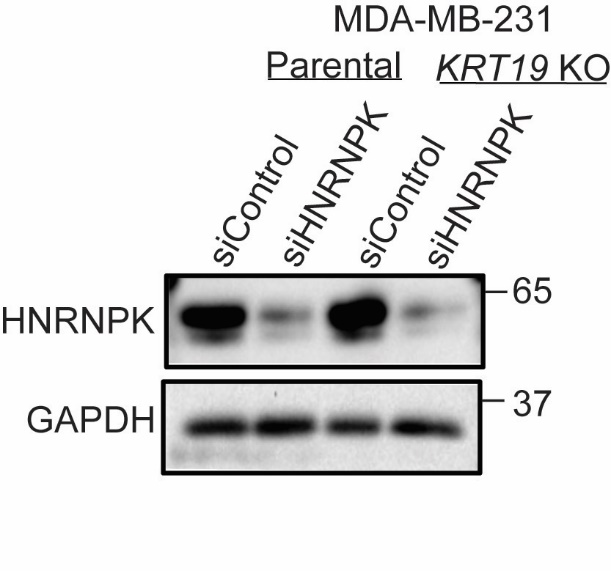


**Supplementary Figure 4.** HNRNPK immunoblot following HNRNPK knockdown. Whole cell lysates of parental control and *KRT19* KO cell lines transfected with siControl or siHNRNPK were harvested, and immunoblotting was performed with antibodies against the indicated proteins.

**
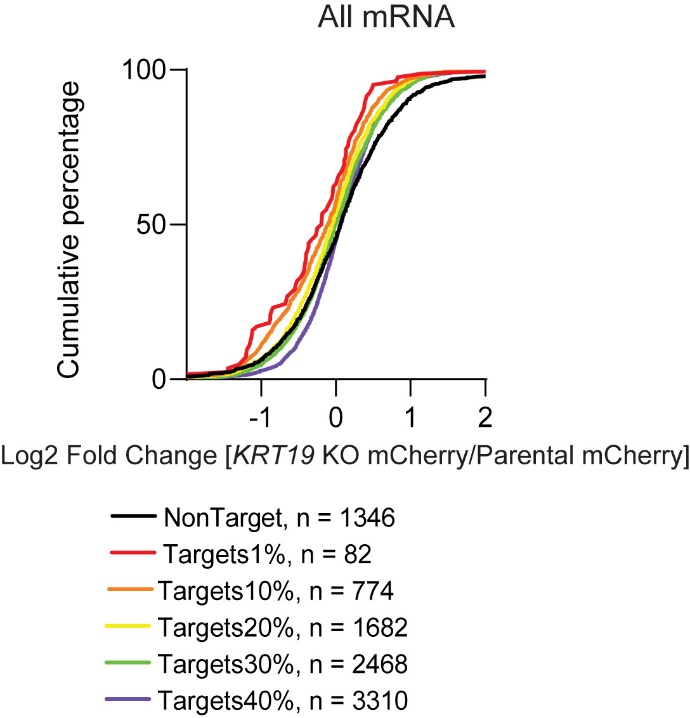
**

**Supplementary Figure 5.** K19 stabilized mRNAs bound to HNRNPK. The empirical cumulative distribution function of mRNA expression changes in parental and *KRT19* KO cells upon mCherry overexpression. HNRNPK targets (colored lines) binned by number of HNRNPK binding sites and non-targets (black line) with minimal gene expression of 4 fragments per kilobase of exon per million mapped fragments ((FPKM) ≥4) are shown.


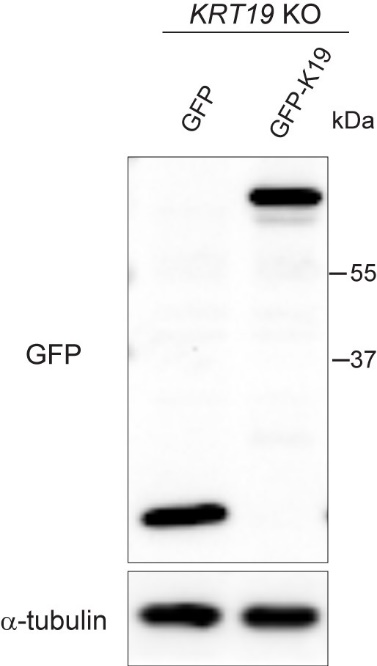


**Supplementary Figure 6.** K19 levels in KO rescue cell lines. Whole cell lysates of *KRT19* KO cell lines stably expressing either GFP or GFP-K19 were harvested, and immunoblotting was performed with antibodies against the indicated proteins.


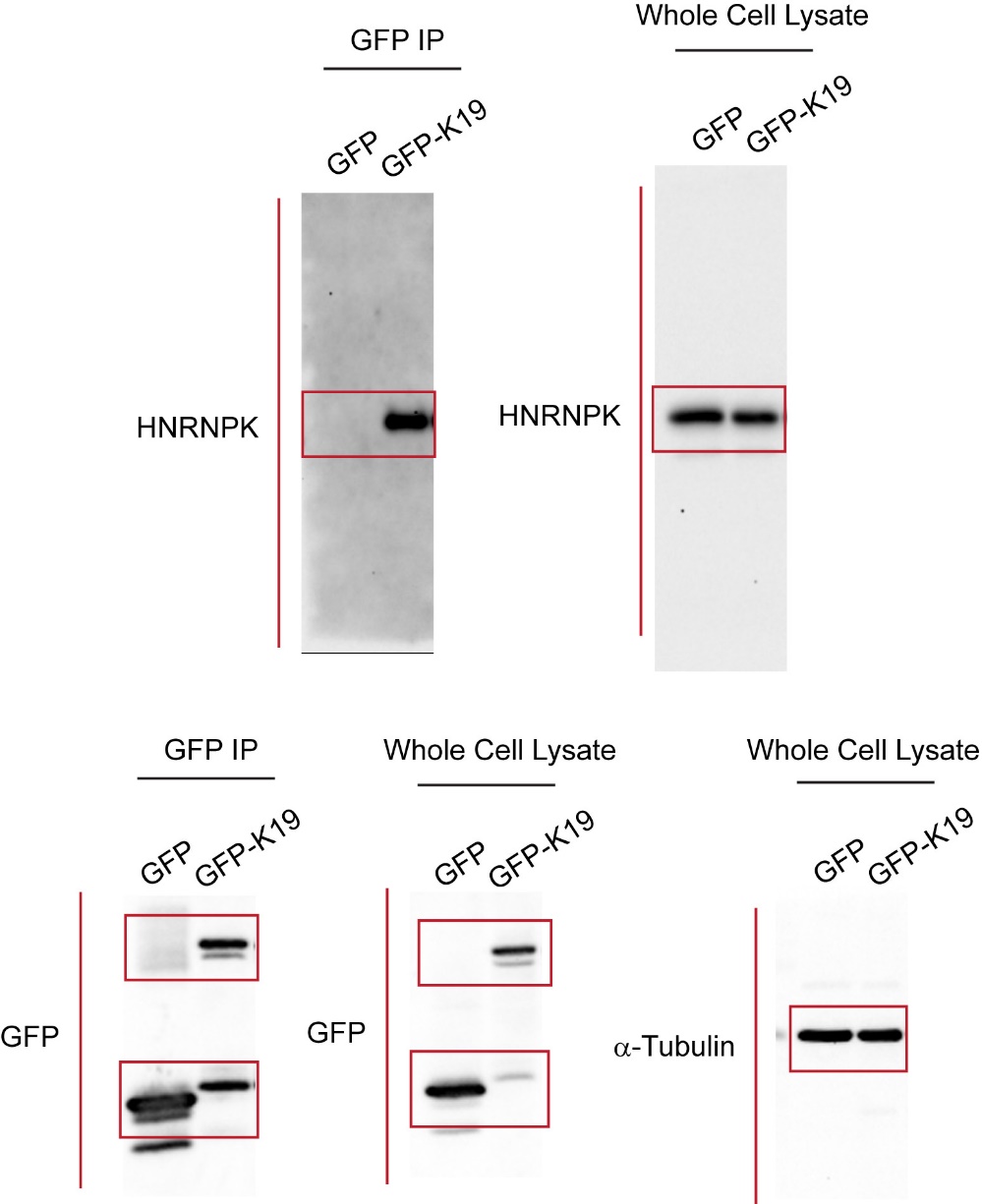


**Supplementary Figure 7**. Full length blots used in Figure 1C. Immunoprecipitated lysates and whole cell lysates from GFP or GFP-K19 overexpressing HEK293 cells were used to run SDS-PAGE gels. Portions of membranes marked with red lines were used for immunoblotting with antibodies against the indicated proteins. The cropped areas used in Figure 1C are shown in red boxes.


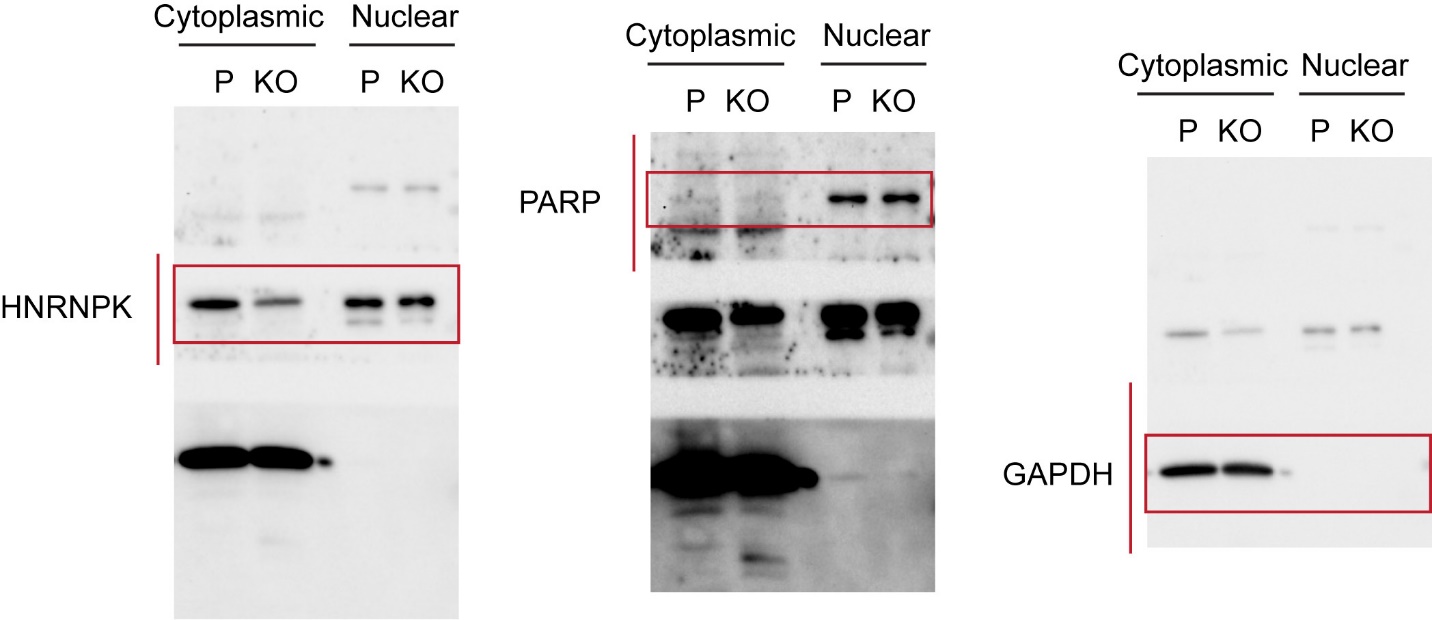


**Supplementary Figure 8**. Full length blots used in Figure 1E. Cytoplasmic and nuclear lysates from parental (P) and *KRT19* KO (KO) cells were used to run SDS-PAGE gels. Portions of membranes marked with red lines were used for immunoblotting with antibodies against the indicated proteins. The cropped areas used in Figure 1E are shown in red boxes.


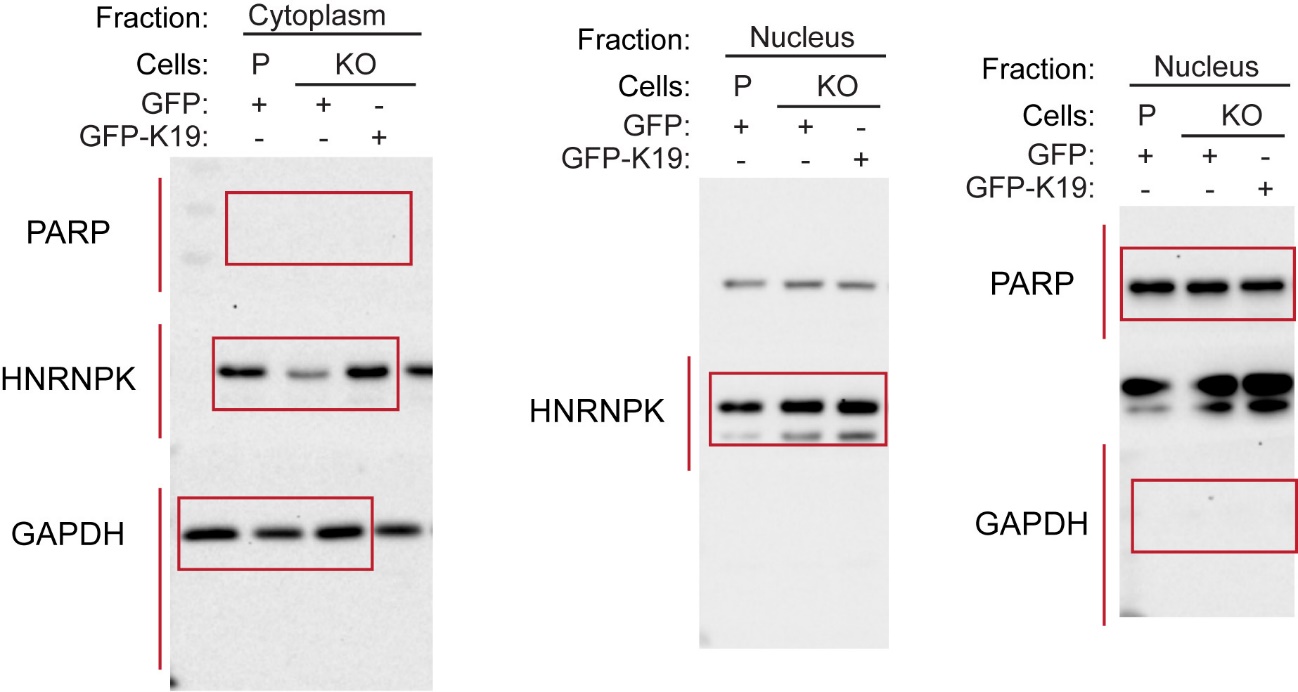


**Supplementary Figure 9**. Full length blots used in Figure 1H. Cytoplasmic and nuclear lysates from GFP or GFP-K19 overexpressing parental (P) and *KRT19* KO (KO) cells were used to run SDS-PAGE gels. Portions of membranes marked with red lines were used for immunoblotting with antibodies against the indicated proteins. The cropped areas used in Figure 1H are shown in red boxes.


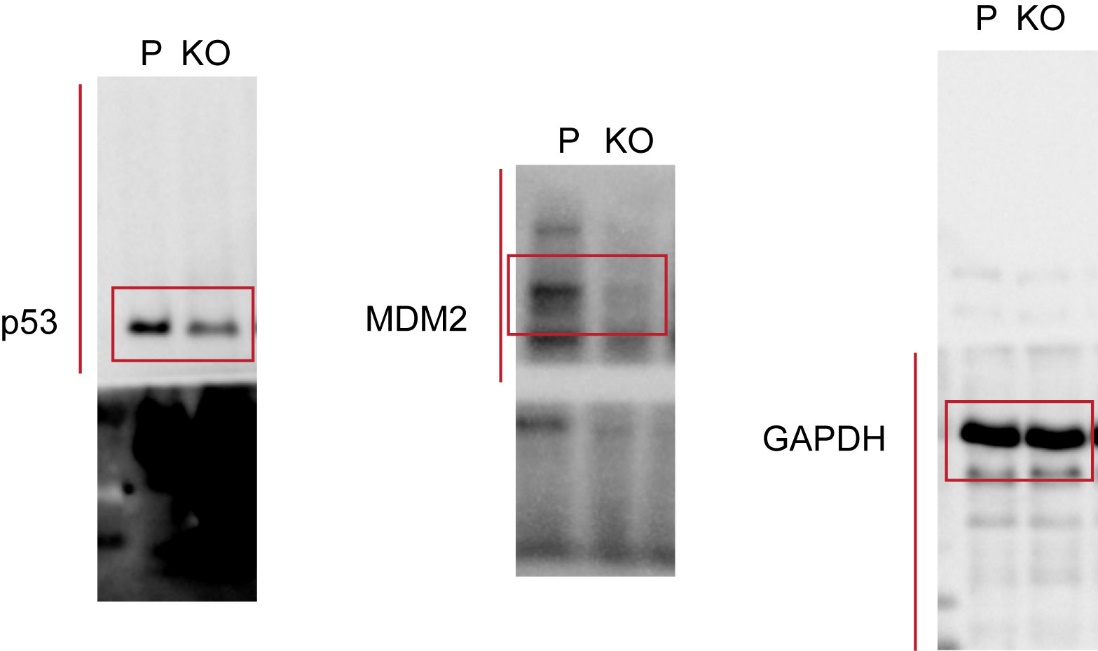


**Supplementary Figure 10**. Full length blots used in Figure 4F. Cell lysates from parental (P) and *KRT19* KO (KO) cells were used to run SDS-PAGE gels. Portions of membranes marked with red lines were used for immunoblotting with antibodies against the indicated proteins. The cropped areas used in Figure 4F are shown in red boxes.


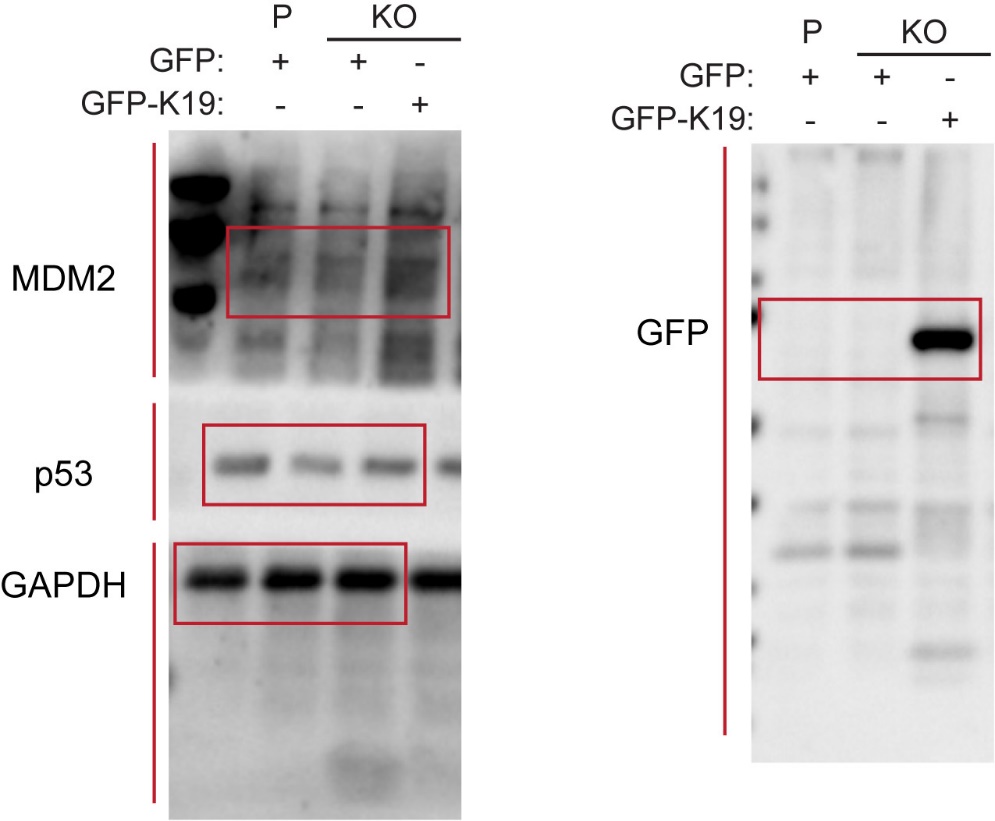


**Supplementary Figure 11**. Full length blots used in Figure 4G. Cell lysates from GFP or GFP-K19 overexpressing parental (P) and *KRT19* KO (KO) cells were used to run SDS-PAGE gels. Portions of membranes marked with red lines were used for immunoblotting with antibodies against the indicated proteins. The cropped areas used in Figure 4G are shown in red boxes.

**Supplementary Tables**

**Supplementary Table 1.** RNA-sequencing result from parental and *KRT19* KO cells.

**Supplementary Table 2.** Cytoplasmic HNRNPK PAR-CLIP result from parental cells

**Supplementary Table 3.** Cytoplasmic HNRNPK PAR-CLIP result from *KRT19* KO cells.

**Supplementary Table 4.** RNA-sequencing result from *KRT19* KO cells transfected with HNRNPK ∆NLS or vector control.

**Supplementary Table 5.** RNA-sequencing result from parental cells transfected with HNRNPK ∆NLS or vector control.

**Supplementary Table 6.** RNA-sequencing result from parental cells transfected with HNRNPK shRNA or vector control.

**Supplementary Table 7.** Combined result of RNA-sequencing result from parental cells transfected with HNRNPK shRNA or vector control and cytoplasmic HNRNPK PAR-CLIP result from parental cells.

**Supplementary Table 8.** Combined result of RNA-sequencing result from parental and *KRT19* KO cells and cytoplasmic HNRNPK PAR-CLIP result from parental cells.
